# Giant pedunculated hemangioma of the liver

**DOI:** 10.1590/0100-3984.2014.0057

**Published:** 2016

**Authors:** Paula de Castro Menezes Candido, Izabela Machado Flores Pereira, Breno Assunção Matos, Mario Henrique Giordano Fontes, Teófilo Eduardo de Abreu Pires, Petrônio Rabelo Costa

**Affiliations:** 1 Hospital Felício Rocho, Belo Horizonte, MG, Brazil.

*Dear Editor,*

**Figure 1:**
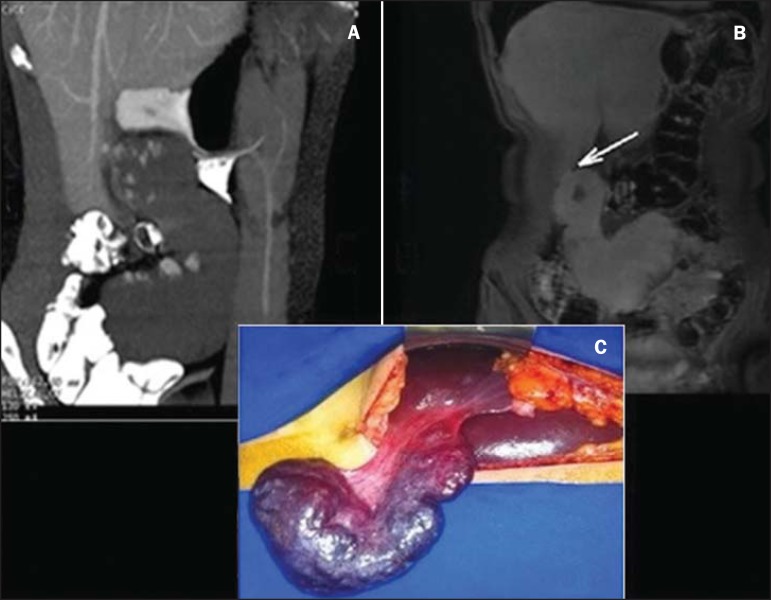
**A:** Contrast-enhanced total abdominal CT (oral and intravenous contrast-enhancement), sagittal section, venous phase showing a well defined mass in the right hypochondrium/flank in continuity with the liver, presenting with a pattern of peripheral, globuliform and centripetal enhancement, with a thin pedicle originating from the segment V. **B:** Coronal MRI, T1-weighted SPGR, at delayed phase showing homogenization of the lesion and identifying a pedicle contiguous with the liver parenchyma (arrow). **C:** Surgical specimen of the reddish blue pedunculated lesion with cirrhotic appearance, showing pedicle contiguous with the liver parenchyma.

A previously healthy, 28-year-old woman presenting a palpable mass in the right hypochondrium for 3 years, evolving with local discomfort over the last 20 days. Ultrasonography (US) demonstrated an expansile mass best characterized by computed tomography (CT) and magnetic resonance imaging (MRI) which showed a well defined solid mass in continuity with the liver by a thin pedicle originating from the segment V and caudally extending towards the pelvis, measuring 18.0 × 9.4 × 5.2 cm, with features and pattern of enhancement suggestive of hemangioma (Figures 1A and 1B). Surgical resection was the treatment of choice because of the patient's symptoms and the risks of torsion. The anatomopathological analysis confirmed the diagnosis (Figure 1C).

Hemangioma is the most common benign liver tumor^(1-8)^, with a prevalence of 0.4-20% in necropsies^(1,5-8)^. In most cases, hemangiomas are small, asymptomatic and incidentally found at imaging studies^(1,2,5,6)^.

In spite of the lack of consensus about the dimensions to define a giant hemangioma, ranging from 4 to 10 cm according to the literature, it is known that the exophytic presentation, particularly those pedunculated, are very rare ^(1-3,5,6)^. The first case was reported by Ellis et al. in 1985; and up to 2013, only 24 cases were described in the literature^(1,4)^.

In almost 50% of cases, pedunculated hemangiomas are symptomatic at the diagnosis^(1)^ and, likely any giant lesion, may determine compression of the intrahepatic biliary ducts, vascular structures or adjacent organs, manifesting with pain, early satiety, hemorrhage, jaundice, nausea and vomiting^(1,2,5,6,8)^. Main complications include torsion due to a long and mobile pedicle, infarction^(5,6)^, spontaneous or traumatic rupture, congestive heart failure, and Kasabach-Merritt syndrome^(2,6,7)^.

A correct diagnosis of the pedunculated lesion may be difficult, despite the typical radiological presentation, because of the limitation in define the origin of the mass, since a thin pedicle may be almost undetectable at images^(1,4,5)^.

The most used modalities of imaging in diagnosis include US, CT and MRI^(1-4,6,8)^. At US, the image is typically hyperechoic, homogeneous, with well defined margins; and, in cases of giant lesions, central heterogeneity may be present^(8)^. At CT, with a certain frequency, giant hemangiomas do not present with the typical pattern of hypoattenuating lesion with centripetal enhancement and homogenization at delayed sections, due to the presence of avascular areas of necrosis, fibrosis or hemorrhage^(3,8)^. MRI is the most sensitive and specific (> 90%) diagnostic method^(4,6)^. The lesions are well defined, homogeneous, with low signal intensity at T1-weighted sequences, and high signal intensity at T2-weighted sequences.

Biopsy is not recommended in such cases, due to the risk of hemorrhage^(6)^.

There are reports in the literature describing pedunculated hemangiomas as gastric, adrenal tumor^(1,4)^, retroperitoneal mass^(1)^, other pedunculated liver tumors such as hepatocellular carcinoma, mesenchymal hamartoma, focal nodular hyperplasia or adenoma^(4)^.

Surgical treatment is reserved for cases of giant or symptomatic lesions, uncertain diagnosis, lesions with complications^(1,2,4-7)^, and for cases of pedunculated hemangiomas due to their tendency to torsion^(5,6)^.
